# Synchronous Retrograde Replantation Method for the Repair of Completely Severed Toes: A Four‐Year Follow‐Up

**DOI:** 10.1002/ccr3.70808

**Published:** 2025-09-11

**Authors:** Qianheng Jin, Lei Xu, Jihui Ju, Ruixing Hou, Yuefei Liu

**Affiliations:** ^1^ Suzhou Ruihua Orthopedic Hospital Suzhou Jiangsu China

**Keywords:** amputation, microsurgery, replantation, toe injuries, treatment outcome

## Abstract

Five‐toe traumatic amputation was successfully managed with synchronous retrograde replantation. Meticulous vascular anastomosis achieved immediate revascularization. All replanted toes survived completely, with excellent functional and cosmetic outcomes maintained at 4‐year follow‐up. This technique demonstrates efficacy for multiple digital amputations despite its technical complexity.

## 1 Introduction

By exerting active pressure on the ground to supplement the body's passive pressure, the toes maintain balance during walking [1, 2]. The great toe plays a dominant role in bearing the load of the forefoot and supports up to 40% of the body weight during walking. As industrialization continues to advance, toe amputations due to industrial‐ and transportation‐related traumas are becoming increasingly prevalent [3, 4]. Owing to limited blood supply and constraints of surgical positioning, the survival rates of toe replantation are notably lower than those of finger replantation. The amputation of five toes poses an especially challenging surgical task. Herein, we introduce a synchronous retrograde replantation technique that reconstructs the five toes in a sequential manner. The procedure involves debridement, suturing the plantar skin, suturing the flexor digitorum tendons, anastomosing the plantar digital arteries and nerves, reduction and fixation of the phalangeal bones, suturing the extensor digitorum tendons, anastomosing the dorsal digital vein, and suturing the dorsal digital skin. This method can quickly restore blood supply to each toe, thereby improving the survival rate of each toe.

## 2 Case History

A 53‐year‐old male patient presented to the hospital with traumatic severance of all five toes on his left foot following a workplace injury when a steel beam fell on his left foot approximately 30 min prior to presentation. The big toe was severed distally within the proximal phalanx, while the 2nd to 5th toes were severed proximally within their respective proximal phalanges. Only the flexor digitorum tendons of all five toes were connected, with severe skin contusion at the site of injury. This patient had no associated injuries in other parts of his body and was in good health prior to the injury. He was neither diabetic nor hypertensive and had no history suggestive of peripheral vascular disease. The patient had been a smoker for 20 years, smoking 10 cigarettes per day; however, he had no history of alcohol abuse.

## 3 Treatment

**FIGURE 1:**
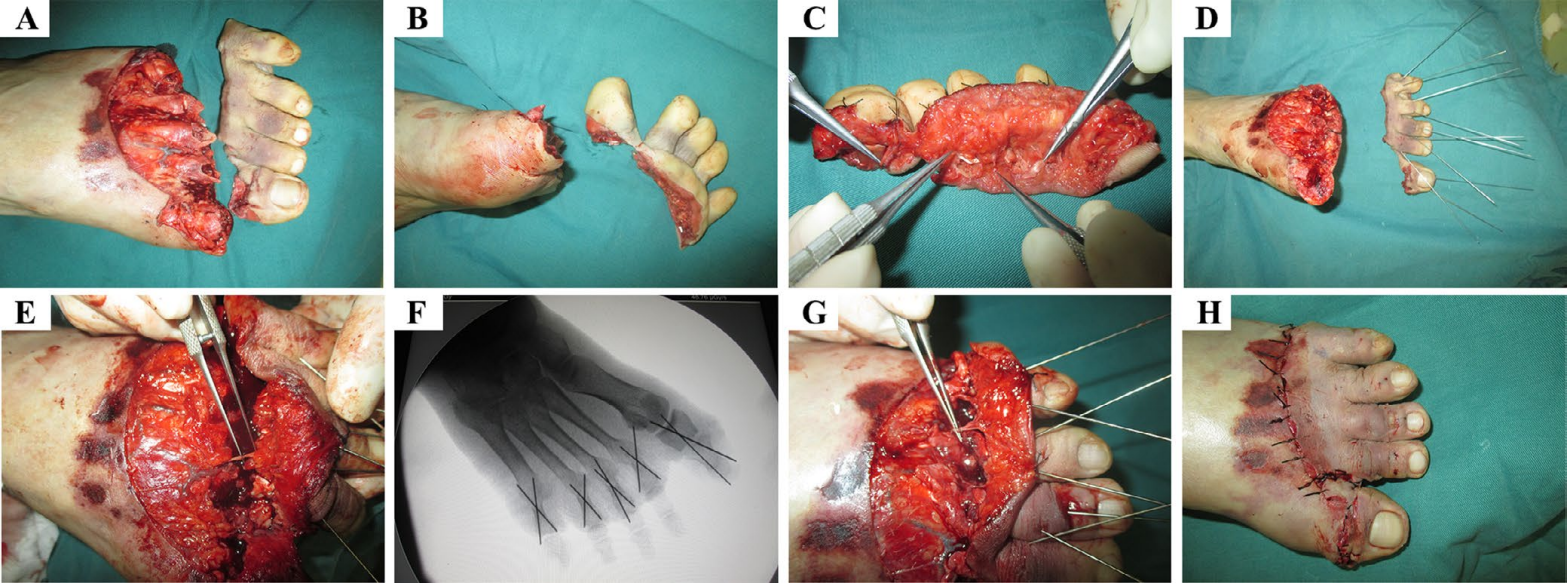
Process of surgery. (A) Dorsal view severed toes (B) Metatarsal view severed toes (C) Vessel mark (D) Kirschner wires retrogradely inserted (E) Arterial anastomosis (F) Fracture fixation (G) Venous anastomosis (H) Skin suturing.

The injured foot was lightly compressed and bandaged for hemostasis. After admission, preoperative examinations were completed, and emergency surgery was performed under combined lumbar and epidural anesthesia. The connected long flexor tendons of the toes were divided to completely sever D1–D5 of the left foot; this was done to facilitate subsequent surgery. The surgery was conducted by two teams during the debridement phase: one team focused on debriding and neurovascular tagging of the distal ends of the severed toes, while the other team simultaneously managed the proximal ends. After completing debridement, a single surgical team proceeded with the subsequent procedures. Two 0.8–1.2 mm Kirschner wires were inserted retrogradely into the fractured end of the severed toes, laying the foundation for subsequent fracture fixation. The replantation procedure was performed in the following sequence: debridement, suturing of the plantar skin, suturing of the flexor digitorum tendons, anastomosing the proper digital arteries and nerves, reduction and fixation of the phalanx, suturing of the extensor digitorum tendon, anastomosing the dorsal digital vein, and suturing of the dorsal digital skin. Blood supply was restored to all toes promptly, and the duration of ischemia was minimized. During the surgery, 10 arteries and 8 veins were anastomosed (two veins in the big toe were sutured separately, while the skin of toes D2–D5 was approximated, allowing a total of 6 veins to be sutured). The ischemic time for toes D1–D5 was 12 h, and the surgery lasted 19 h. After the procedure, the blood flow to toes D1–D5 was excellent (Figure 1).

## 4 Results

**FIGURE 2:**
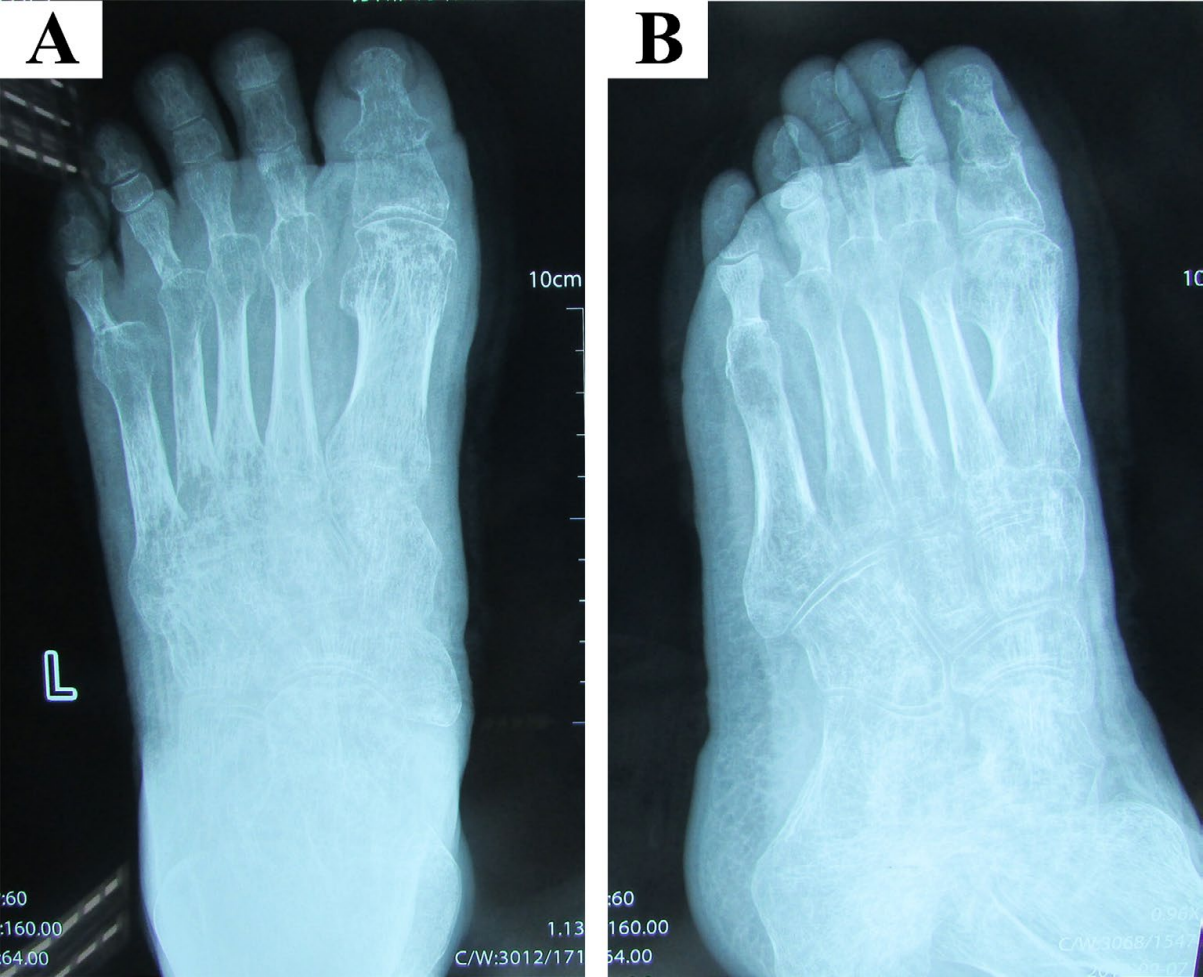
Four months postoperatively, the fracture had fully healed. (A) Routine frontal X‐ray (B) Oblique X‐ray.

**FIGURE 3:**
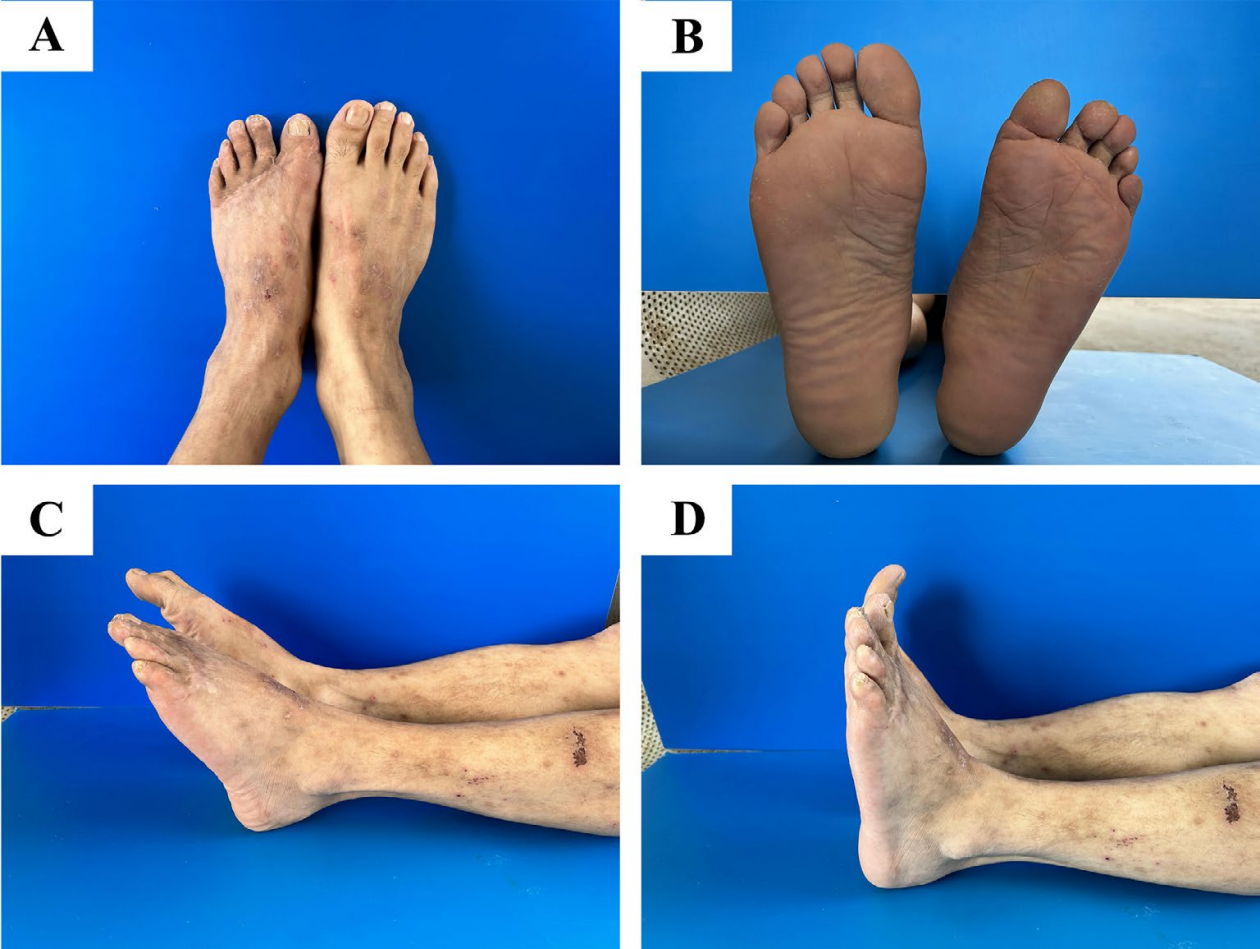
Appearance at 4 years postoperatively. (A) Dorsal view (B) Metatarsal view (C) Plantar flexion position (D) Dorsiflexion position.

Two months postoperatively, the scab shed naturally, the wound healed adequately, and the fractured bones united appropriately. The Kirschner wires were subsequently removed (Figure 2). Four months postoperatively, the fractures had fully healed (Figure 3). Four years postoperatively, the patient regained two‐point discrimination in D1–D5 toes and regained an adequate range of active flexion and extension of the left ankle joint (Video S1). The patient had no significant limitation in walking (Video S2). The patient achieved a postoperative Maryland Foot Function Score of 89 at 4 years postoperatively, as calculated from standardized assessments of pain (20/20), function (50/55), cosmesis (19/25).

## 5 Discussion

The feet primarily bear the weight of the human body [5]. Amputations of multiple digits, particularly injuries to the proximal interphalangeal and metatarsophalangeal joints, significantly affect the arch and load‐bearing capacity of the foot. Therefore, if a patient can tolerate surgery and the toes are intact, multiple digit injuries should be replanted, and efforts should be made to ensure their survival. However, toe replantation poses a significant challenge even to skilled microsurgeons [6]. In 2008, Lin et al. reviewed 20 surgeries for replantation of the great toe. The toes survived in 11 cases, while nine cases failed, making an overall survival rate of 55%. The primary causes of failure were arterial insufficiency and vascular spasm. Lin et al. suggested that due to the lower survival rate, replantation of the great toe should be limited to traumatic dislocations in children and incomplete dislocations in adults, especially amputations at the proximal interphalangeal joint [7]. With continuous advancements in technical proficiency, the experience of surgeons, and surgical instruments, the survival rates of toe replantation have gradually improved [8, 9]. In 2022, Cui et al. conducted a review of 10 cases of toe replantation, encompassing a total of 13 toes. Only one case of toe necrosis occurred, resulting in a survival rate of 92.3%. This survival rate was almost equivalent to that of finger replantation. In the sample case, toes D2–D5 were severed in a female patient. By reconstructing both plantar arteries and 2–3 dorsal veins in each toe, the severed toes survived postoperatively. During the 3‐year postoperative follow‐up, there was no significant difference in growth between the replanted toes and the contralateral toes, and this alleviated the patient's psychological concerns as she could confidently use high‐heeled shoes and expose her toes [10]. Therefore, successful toe replantation not only preserved foot function but was also psychologically beneficial to the patient.

Although the survival rate of toe replantation has improved, there is still a significant risk of necrosis. Free flap transplantation has been used effectively as a remedial measure in cases of necrosis. Lee et al. applied a medial sural artery perforator flap to repair necrosis of toes D1–D3 after replantation surgery. This flap had the characteristics of being modernized, aesthetic, small, thin, and pliable was associated with minimal damage to the donor site. Through subsequent surgeries, including splitting and degreasing, the foot gained a cosmetically satisfactory appearance. However, the severed toe lost its nail [11]. Herein, we proposed a synchronous retrograde replantation method for the treatment of cases of multi‐toe amputation based on the original foundation. Retrograde replantation could reduce the difficulty of anastomosing arteries and improve the quality of vascular anastomosis. Our synchronous method could also quickly restore arterial blood supply to each toe, thereby reducing the ischemic time. Furthermore, for the management of fractured ends, we utilized conventional shortening and implemented cross‐fixation to ensure joint mobility. Synchronous retrograde replantation could significantly improve operative success rates and foot function in patients with multi‐toe replantation.

## 6 Conclusion

Surgical replantation of severed toes is quite challenging. Our synchronous retrograde replantation technique could significantly improve the success rate and foot function of patients after multi‐toe replantation.

## Author Contributions

**Qianheng Jin:** conceptualization, data curation, formal analysis, investigation, writing – original draft. **Lei Xu:** data curation, formal analysis, investigation, software. **Jihui Ju:** data curation, project administration, supervision. **Ruixing Hou:** conceptualization, supervision, writing – review and editing. **Yuefei Liu:** conceptualization, funding acquisition, writing – review and editing.

## Consent

Written patient consent has been signed and obtained from the patient.

## Conflicts of Interest

The authors declare no conflicts of interest.

## Supporting information


**Video S1.** Foot Exercise Conditions at 4 years postoperatively.

**Video S2.** Walking conditions at 4 years postoperatively.

## Data Availability

The data that support the findings of this study are available from the corresponding author upon reasonable request.
